# Alpha-l-Fucosidase Isoenzyme iso2 from *Paenibacillus thiaminolyticus*

**DOI:** 10.1186/s12896-015-0160-x

**Published:** 2015-05-27

**Authors:** Eva Benešová, Petra Lipovová, Jana Krejzová, Terezia Kovaľová, Patricie Buchtová, Vojtěch Spiwok, Blanka Králová

**Affiliations:** Department of Biochemistry and Microbiology, UCT Prague, Prague, 166 28 Czech Republic

**Keywords:** α-l-fucosidase, *Paenibacillus thiaminolyticus*, Transglycosylation, Carbohydrate active enzymes, Glycosidase

## Abstract

**Background:**

α-l-Fucosidases are enzymes involved in metabolism of α-l-fucosylated molecules, compounds with a fundamental role in different life essential processes including immune response, fertilization and development, but also in some serious pathological events. According to the CAZy database, these enzymes belong to families 29 and 95. Some of them are also reported to be able to catalyze transglycosylation reactions, during which α-l-fucosylated molecules, representing compounds of interest especially for pharmaceutical industry, are formed.

**Methods:**

Activity-based screening of a genomic library was used to isolate the gene encoding a novel α-L-fucosidase. The enzyme was expressed in E.coli and affinity chromatography was used for purification of His-tagged α-L-fucosidase. Standard activity assay was used for enzyme characterization. Thin layer chromatography and mass spectrometry were used for transglycosylation reactions evaluation.

**Results:**

Using a genomic library of *Paenibacillus thiaminolyticus*, constructed in *E.coli* DH5α cells, nucleotide sequence of a new α-l-fucosidase isoenzyme was determined and submitted to the EMBL database (HE654122). However, no similarity with enzymes from CAZy database families 29 and 95 was detected. This enzyme was produced in form of histidine-tagged protein in *E.coli* BL21 (DE3) cells and purified by metaloaffinity chromatography. Hydrolytic and transglycosylation abilities of α-l-fucosidase iso2 were tested using different acceptor molecules.

**Conclusions:**

In this study, new enzyme α-l-fucosidase iso2 originating from *Paenibacillus thiaminolyticus* was described and prepared in recombinant form and its hydrolytic and transglycosylation properties were characterized. As a very low amino acid sequence similarity with known α-l-fucosidases was found, following study could be important for different biochemical disciplines involving molecular modelling.

**Electronic supplementary material:**

The online version of this article (doi:10.1186/s12896-015-0160-x) contains supplementary material, which is available to authorized users.

## Background

Saccharides and different types of glycoconjugates constitute a diverse group of molecules, function of which range from both energy and building elements supply to facilitation of specific interactions and to modifications of properties and functions of many biologically active molecules [1, 2]. Many enzymes are involved in metabolic pathways of these molecules. Two different systems of classification were evolved for glycosidases, which are responsible for hydrolytic cleavage of glycosidic bond. First of them, coordinated by Nomenclature Committee of the International Union of Biochemistry and Molecular Biology, is based on substrate specificity of particular enzymes [3]. Unfortunately, broader substrate specificity of some glycosidases can be taken into account by assigning of more EC numbers to one enzyme only. Second classification is represented by Carbohydrate-Active Enzymes database (CAZy), which is based on amino acid sequence similarities of particular enzymes [4, 5, 6].

α-l-Fucosidases (3.2.1.51) are glycosidases that are able to cleave terminal α-l-fucosyl moiety from different types of oligosaccharides and glycoconjugates. According to Carbohydrate-Active Enzymes database (CAZy database), they belong to families 29 and 95, which differ among each other in mechanism used for the hydrolytic reaction catalysis [7]. Family 29 enzymes use a two-step double-displacement mechanism for the catalysis, (retaining enzymes i.e. the anomeric configuration of the released product is the same as in the originally cleaved molecule), while glycosidases from family 95 are among so called inverting enzymes catalyzing the hydrolytic process by direct displacement mechanism. Many retaining enzymes are able to catalyze not only hydrolytic reactions but also transglycosylation; i.e. the reaction, in which the final acceptor of cleaved glycosidic residue is a hydroxyl group-possessing molecule that differs from water [7, 8, 9, 10].

α-l-Fucosidases catalyzing transglycosylation reactions might be interesting in synthesis of α-l-fucosylated molecules with potential application especially in pharmaceutical industry, as these molecules are involved in many life-important or life-threatening processes in eukaryotic organisms. Cell differentiation, development, fertilization, embryogenesis, angiogenesis, apoptosis, inflammation or prevention of host-pathogen interaction are examples of natural processes, in which α-l-fucosylated molecules play a crucial role. ABO blood group antigens are also α-l-fucosyl moiety containing molecules. Cancer, fucosidosis, infection by pathogens, leukocyte adhesion deficiency type II, rheumatoid arthritis or cystic fibrosis represent pathological events closely associated with α-l-fucosylation. In some cases, the defect in their metabolism is the nature of the disease (e.g. fucosidosis), in other cases α-l-fucosylated motifs play an important role in the disease propagation (e.g. cancer metastasis). In several cases, changes of α-l-fucosylation were observed but the particular connection with the disease has not yet been clarified. In the pharmaceutical industry synthesis of α-l-fucosylated molecules could be used e.g. for preparation of down-regulating inflammatory response therapeutics or for targeted production of antibodies. Drugs for antiadhesion therapy, cancer vaccines and transplantology could be other areas for α-l-fucosylated molecules application. Fucosylated oligosaccharides could be also used in the food and cosmetic industries, as surface active agents helping to reduce surface tension and increase the solubility of otherwise insoluble materials [11, 12, 13, 14, 15, 16, 17].

The aim of this study was to confirm the presence of a second enzyme of α-l-fucosidase in the bacterial strain *P. thiaminolyticus* and to characterize its hydrolytic and transglycosylation abilities. During the study we constructed a genomic library of *P. thiaminolyticus* in *E.coli* DH5α cells and detected nucleotide sequence of a new α-l-fucosidase iso2. Consequently, we prepared an expression plasmid (pET16b-αLF2) and produced the enzyme as histidine-tagged fusion protein in *E.coli* BL21 (DE3) cells. Hydrolytic and transglycosylation abilities (using different acceptor molecules) were tested for the enzyme purified by metaloaffinity chromatography.

## Results and discussion

### Isoenzymes detection

During initial experiments, while testing the possibility to purify an enzyme with α-l-fucosidase activity by chromatographic methods, it was accidentally discovered that *P. thiaminolyticus* produces two isoenzymes of α-l-fucosidase and that it is possible to separate them using chromatography with hydrophobic interactions on the column HiTrap Butyl FF (1 mL) (data not shown). This discovery was confirmed by native electrophoresis in polyacrylamide gel followed by staining with chromogenic substrate *p*-nitrophenyl-α-l-fucopyranoside (*p*NPα-l-Fuc). Both enzymes with α-l-fucosidase activity displayed different mobility. Cloning, sequencing and characterization of hydrolytic and transglycosylation activity of α-l-fucosidase isoenzyme iso1 was described in Benešová et al 2013 [18]. Results obtained for α-l-fucosidase isoenzyme iso2 (numbering used according to the order of nucleotide sequence discovery) will be reported here.

At first, genomic library constructed from chromosomal DNA fragments of *P. thiaminolyticus* and plasmid pUC19 in *E.coli* DH5α cells was screened for colonies displaying α-l-fucosidase activity. The simplicity of the screening was supported by the fact that cells of *E.coli* DH5α don’t posses any α-l-fucosidase activity. Plasmid DNA of the colony with the ability to cleave 5-bromo-4-chloro-3-indolyl α-l-fucopyranoside (X-Fuc) was isolated and sequenced by the method of primer walking (Geneart). The results of sequencing confirmed the presence of a gene encoding the enzyme with another sequence than formerly identified gene for α-l-fucosidase iso1. The start of the gene was identified by a determination of a presumable Shine-Dalgarno sequence 5’-AGGAGGA-3’preceding the initial methionine. Product of this newly identified gene will be designed as α-l-fucosidase iso2 in the following text. Interestingly, the identified Shine-Dalgarno sequence was slightly different from the sequence, which was found for α-l-fucosidase iso1, i.e. 5’–AAGGAGAGAA–3’. This fact probably indicates different roles of both isoenzymes in *P.thiaminolyticus*, as it is known that different Shine-Dalgarno sequences are responsible for regulation of gene expression and of protein synthesis [18, 19]. The determined nucleotide sequence of α-l-fucosidase iso2 was submitted to the EMBL database and is available under the Accession No HE654122. The identified sequence of α-l-fucosidase iso2 was also compared with known protein sequences using WU-BLAST2 program. Interestingly, majority of the results referred only to hypothetical bacterial proteins, function of which was not experimentally confirmed. This low similarity of α-l-fucosidase iso2 with known enzymes, especially with glycosidases with α-l-fucosidase activity, could represent an interesting field of study for molecular modelling. Preliminary modelling studies showed that α-l-fucosidases iso1 and iso2 have a common TIM-barrel protein fold and retaining mechanism of action. α-l-Fucosidase iso2 exhibits low homology with the 42 and 29 families (α-l-fucosidase iso 1 being a member of the family 29), the domain organization refers rather to the family 42.

### Recombinant protein production

**Fig. 1:**
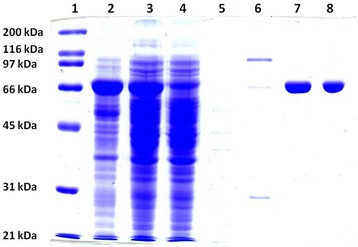
Analysis of the expression and purification of the recombinant α-l-fucosidase from *P. thiaminolyticus* by SDS-PAGE. Electrophoresis was carried out in 10 % polyacrylamide gel and proteins were visualized by Coomassie Brilliant Blue R-250. Line 1- SDS-PAGE Molecular Weight Standards, Broad Range, line 2 - cells of *E.coli* BL21 (DE3) after expression of α-l-fucosidase iso2, line 3 - supernatant after disintegration of cells after expression, line 4 - proteins which did not bind to the Ni-NTA agarose after the application of the supernatant sample, line 5 - fraction after wash in the binding buffer with 10 mM imidazole, line 6 - fraction after wash in the binding buffer with 40 mM imidazole, lines 7 and 8 - recombinant α-l-fucosidase iso2 from *P. thiaminolyticus* eluted by the binding buffer with 250 mM imidazole and desalted by gel filtration on the column PD10

The recombinant α-l-fucosidase iso2 expressed as a fusion protein with polyhistidine tag has a molecular weight of 76.8 kDa. The target protein was gained by affinity chromatography on the column with Ni-NTA agarose, exploiting the interaction of Ni^2+^ ions and fusion histidine tag of the isolated protein. Protein concentration in obtained samples of α-l-fucosidase iso2, stained by the Bradford protein assay, was approximately 1 mg/mL. Processes of cultivation, disintegration and purification were followed by sodium dodecyl sulfate polyacrylamide gel electrophoresis (SDS-PAGE) (Fig 1). Only one single band, corresponding to the estimated molecular weight, is visible after the staining by Coomassie Brilliant Blue R-250 in samples after the affinity chromatography.

### Characterization of hydrolytic activity

Purified enzyme was used for the characterization of its hydrolytic activity with *p*NPα-L-Fuc as a substrate. Specific activity of used samples was approximately 30 μmol/min/mg. Standard experiments for determination of a temperature profile, pH optimum and the dependence of initial velocity on the concentration of *p*NPα-L-Fuc in a reaction mixture were carried out. The enzyme exhibited the highest activity at 50 °C (under chosen conditions and a reaction time of 10 min) and the pH of 6.5 was confirmed as optimum. Kinetic parameters are summarized in Table 1 together with characteristics of α-l-fucosidase iso1. Interestingly, while measuring the dependence of initial velocity on the concentration of *p*NPα-L-Fuc, both isoenzymes exhibited very similar behaviour caused very probably by substrate inhibition of the enzyme at high concentrations of *p*NPα-L-Fuc. This phenomen could be a result of simultaneous binding of more substrate molecules into the active site, thus loosing the correct orientation for the process of hydrolysis. Interestingly, the values of K_M_ and K_S_ are very similar for both isoenzymes, although α-l-fucosidase iso2 doesn’t belong (according to the amino acid sequence) to the same glycosidase family as α-l-fucosidase iso1. α-l-Fucosidase iso2 achieves almost twice the enzyme efficiency of α-l-fucosidase iso1.

**Table 1:** Comparison of kinetic parameters of α-l-fucosidase isoenzymes from *P. thiaminolyticus* using chromogenic substrate *p*NPα-l-Fuc

	α-L-fucosidase	
	iso1	iso2
K_m_	0.44 ± 0.02 mmol/L	0.52 ± 0.05 mmol/L
K_s_	83 ± 8 mmol/L	79 ± 20 mmol/L
k_cat_	58.7 ± 0.8 s^-1^	111 ± 3 s^-1^
k_cat_/K_m_	133 ± 7 (mmol/L)^-1^ s^-1^	213 ± 30 (mmol/L)^-1^ s^-1^

The role of two isoenzymes of α-l-fucosidase in a prokaryotic organism *P. thiaminolyticus* is not clear yet and the answer could be e.g. the substrate specificity of both isoenzymes. Results published by Ashida and coworkers in 2009 could support this hypothesis [20]. The authors identified two isoenzymes of α-l-fucosidase produced by *Bifidobacterium bifidum* JCM1254, belonging to two different glycoside hydrolase families (29 and 95) and differing in substrate specificity (1,2-α-l-fucosidase and 1,3–1,4–α-l-fucosidase). The authors suggested that simultaneous presence of both isoenzymes is essential for the complete degradation of human milk oligosaccharides [20]. Substrate specificity study of both isoenzymes from *P. thiaminolyticus* will be the aim of following experiments.

### Characterization of transglycosylation activity

During designing of transglycosylation experiments a few parameters had to be optimized, e.g. reaction temperature or the use of an organic solvent in a reaction mixture. The solubility of used substrate *p*NPα-l-Fuc (a donor of α-l-fucosyl moiety) was increased by addition of dimethylformamide (DMF). 10 % DMF concentration in a reaction mixture was chosen as the best compromise, as the enzyme possesed almost 80 % of original activity after 6 hours incubation in 10 % DMF and the complete lost of activity was not observed until 24 hours of incubation (measured at 37 °C). Transglycosylation reactions were carried out at 40 °C, as the enzyme was stable at this temperature (70 % of original activity after 20 hours of incubation) and its activity was relatively high. Higher temperatures caused a fast lost of activity (e.g. 0 % activity after 6 hours of incubation at 50 °C) but on the other hand, at lower temperatures the activity of the enzyme was too low for transglycosylation purposes and also solubility of *p*NPα-l-Fuc was not sufficient. Different conditions (e.g. 50 °C) were used for early experiments with *p*-nitrophenyl glycopyranosidic acceptors (for more details see the Methods) to compare transglycosylation abilities of isoenzyme iso1 and iso2 better. However, after low transglycosylation potential of α-l-fucosidase iso2 was confirmed, new experiments with optimized reaction conditions for this enzyme were not carried out.

During transglycosylation experiments different types of molecules were tested as suitable acceptors, i.e. *p*-nitrophenyl glycopyranosides, free saccharides, alcohols and amino acids. All reactions were analyzed by thin layer chromatography (TLC), products containing a saccharidic part were visualized by 2-methylresorcinol. Results of transfucosylation reactions are summarized in Table 2. Data obtained indicate that the enzyme is able to use *p*-nitrophenyl glycopyranosides, free saccharides and alcohols with a very short alkyl chain as acceptors during tranglycosylation reactions. While using methanol as an acceptor of l-fucose, the highest yield was achieved - 15 %. Products of transglycosylation of *N*-acetyl-d-glucosamine, methanol and ethanol were confirmed in reaction mixture (after removal of the enzyme by filtration) by mass spectrometry (data not shown).

**Table 2:** Results of transfucosylation reactions catalyzed by α-l-fucosidase iso2 from *P. thiaminolyticus*

Acceptor	Transglycosylation products	Acceptor	Transglycosylation products
*p*NPα-L-Fuc	+	methanol	+/*
*p*NPα-D-Gal	+	ethanol	+/*
*p*NPα-D-Glc	+	1-propanol	-
*p*NPα-D-Man	+	2-propanol	-
D-glucose	+	butanol	-
D-galactose	+	pentanol	-
D-fructose	+	1-octanol	-
D-mannose	+	Boc-L-Ser-OMe	-
D-glucosamine	+	Boc-L-Thr-OMe	-
*N*-acetyl-D-glucosamine	+/*		
D-maltose	+		

*p*NPα-l-Fuc served as a donor of α-l-fucosyl moiety in all cases

The symbol + indicates that the enzyme was able to use the acceptor for α- l -fucosyl transfer

The symbol - indicates that no transglycosylation product was detected by TLC

The symbol * indicates that the transglycosylation product was confirmed by mass spectrometry

From obtained results it is possible to conclude that α-l-fucosidase iso2 from *P. thiaminolyticus* exhibits significantly lower transglycosylation potential than α-l-fucosidase iso1 from the same origin, as we reported in the publication Benešová et al. 2013 [18]. For comparison, the yields obtained for α-l-fucosidase iso1 during transglycosylation experiments were as follows: *p*NPα-l-Fuc (14 %), *p*NPα-d-Gal (32 %), *p*NPα-d-Glc (21 %), methanol (69 %), ethanol (56 %), 1-propanol (48 %), 2-propanol (34 %), Boc-l-Ser-OMe (3.5 %) and Boc-l-Thr-OMe (3.2 %). However, the ability of α-l-fucosidase iso2 to catalyze transglycosylation reaction may indicate the mechanism of catalysis, i.e. the mechanism with retention of the anomeric configuration. In such a case cleaved glycosyl residue is not transported from the covalent intermediate, generated during the first step of catalytic process, to the molecule of water but to another acceptor molecule with free hydroxyl group [8, 10]. All known α-l-fucosidases using this type of catalysis are classified in CAZy family 29, including the isoenzyme 1 from *P. thiaminolyticus*. However, the above-mentioned low sequence similarity of isoenzyme 2 with known α-l-fucosidases more likely suggests the possibility to classify this enzyme in a new family.

## Conclusion

In this work we identified, cloned, purified and characterized the second isoenzyme of α-l-fucosidase (called α-l-fucosidase iso2) originating from *P. thiaminolyticus*. Comparison of found sequence of α-l-fucosidase iso2 with known sequences of α-l-fucosidases revealed only low similarity and made this newly described enzyme an interesting subject for crystallographic studies and molecular modelling. Experiments testing the ability of the enzyme to catalyze transfucosylation reactions with different types of acceptor molecules showed only low transglycosylation potential in comparison with the α-l-fucosidase iso1 originating from the same microorganism. Further experiments which could help to understand the presence of two isoenzymes in prokaryotic organism will be carried out.

## Methods

### Bacterial strains and cultivation conditions

A strain of *P. thiaminolyticus* (CCM 3599, Czech Collection of Microorganisms in Brno) was cultivated in Luria-Bertani (LB) medium, 16 h, 30 °C, 250 rpm. *E. coli* DH5α (GibcoBRL) and *E. coli* BL21 (DE3) (Novagen) were cultivated in LB medium containing ampicillin (final concentration was 0.1 mg/mL, AppliChem GmbH), 16 h, 37 °C, 250 rpm. After cultivation, bacterial cultures were harvested by centrifugation (4,000 x g, 20 min, 4 °C).

### α-l-Fucosidase isoenzymes Identification

The cell lysate from *P. thiaminolyticus* was prepared using a lysozyme (final concentration of 5 mg/mL, 30 min incubation at a laboratory temperature, Fluka), and a sodium deoxycholate (final concentration of 0.1 %, 30 min incubation at 4 °C, Fluka). Disintegration process was followed by addition of DNaseI (20 U to 1 mL, 15 min incubation at laboratory temperature, Sigma-Aldrich) and sonication (20 W, 6 × 30 s, on ice) with Sonicator® 3000 ultrasonic liquid processor (Misonix Inc.). The insoluble cell debris was removed by centrifugation (20,000 × g, 20 min, 4 °C). The clear supernatant was precipitated using ammonium sulphate. The precipitate between 40 and 75 % of saturation was collected, resuspended in 25 mM EPPS buffer, pH 8.0 containing 750 mM ammonium sulphate and applied onto the column HiTrap Butyl FF (1 mL) (GE Healthcare). The decreasing linear gradient of ammonium sulphate (1 M – 0 M) in 25 mM EPPS buffer, pH 8.0 was used for the elution. The activity of α-l-fucosidase was measured in the collected fractions using chromogenic substrate *p*NPα-l-Fuc and native electrophoresis was used for confirmation of the two isoenzymes presence. The electrophoresis was performed under native conditions (6.8 % running gel, 100 V, 4 °C) and the α-l-fucosidase activity was detected directly in the gel using chromogenic substrate *p*NPα-l-Fuc (6.6 mM in 25 mM EPPS buffer, pH 8.0).

### Identification of the gene for α-l-fucosidase iso2

The gene of α-l-fucosidase iso2 was found in the same procedure as the gene of α-l-fucosidase iso1 from *P. thiaminolyticus* [18] using chromogenic substrate X-Fuc (Biosynth AG®) for screening genomic library in *E. coli* DH5α. Positive colony was found and its plasmid DNA was sequenced by the “primer walking” method (Geneart). The open reading frames were identified using the WU-BLAST2 program.

### *Construction of an expression plasmid* pET16b-αLF2

The gene of α-l-fucosidase iso2 was inserted into the plasmid pET16b (Novagen) containing His-Tag. Plasmid was cleaved by restriction endonucleases *Nde*I and *Xho*I (New England BioLabs), dephosphorylated by Calf Intestinal Alkaline Phosphatase (Invitrogen) and purified by Wizard® DNA Clean-Up System (Promega Corporation). The DNA fragment containing the α-l-fucosidase gene was obtained by polymerase chain reaction (PCR) using primers, in which the *Nde*I restriction site was inserted at the 5’- end of the gene (GACGACGACATATGCGCTACAGACAGGTTCACC) and *Xho*I restriction site at the 3’- end (CCTTCCTCCTCGAGCTACTCATTATACTCTACGACG). The plasmid DNA from positive colony was used as a template. PCR product was purified by a commercial kit Wizard® SV Gel and PCR Clean-Up System (Promega Corporation). The purified DNA fragment was treated with *Nde*I and *Xho*I endonucleases, and ligated using T4-DNA ligase (New England BioLabs) to the linearized and dephosphorylated expression plasmid pET16b. The competent cells of *E. coli* DH5α were transformed [21] by the ligation mixture and cultivated on LB plates with ampicillin. Plasmid DNA of the arised colonies was screened by restriction endonucleases, and the gene of α-l-fucosidase iso2 in the positive colony was confirmed by sequencing. The name pET16b-αLF2 is used for this construct in the text.

### Expression and purification of recombinant α-l-fucosidase iso2

The cells of *E.coli* BL21 (DE3) were used for production of recombinant α-l-fucosidase iso2. These cells transformed by pET16b-αLF2 were grown at 37 °C in LB medium containing ampicillin (final concentration 0.1 mg/mL). Isopropyl β-d-thiogalactopyranoside (IPTG) as an inducer of expression was added after 5 hours of cultivation (OD_600nm_ 0.6) to the final concentration 300 μmol/L. The α-l-fucosidase iso2 was expressed for another 4 hours. The cells were harvested by centrifugation (6,000 x g, 15 min, 4 °C).

The bacterial pellet was resuspended in binding buffer (50 mM phosphate buffer, pH 7.0, 150 mM KCl) and disintegrated in the same way as the cells of *P. thiaminolyticus*. The insoluble cell debris was removed by centrifugation (20,000 × g, 20 min, 4 °C) and the clear supernatant was stored at –20 °C or applied directly to Ni-NTA Agarose (Novagen) preequilibrated with the binding buffer with 10 mM imidazole. The standard purification protocol for Ni-NTA Agarose was used for purification of α-l-fucosidase iso2. The concentration of imidazole in binding buffer was 40 mmol/L for washing and 250 mmol/L for elution. After elution all fractions were assayed for α-l-fucosidase activity. Fractions displaying α-l-fucosidase activity were put together and desalted using gel chromatography (PD10, GE Healthcare).

SDS-PAGE was used for molecular weight and purity determination. It was performed in 10 % polyacrylamide gel in a Mini-Protean III dual-slab cell electrophoresis unit (Bio-Rad) under reducing conditions [22] and the proteins were visualized by Coomassie Brilliant Blue R250.

### α-l-Fucosidase activity assay

The hydrolytic activity of α-l-fucosidase was determined by measuring the release of *p*-nitrophenol from *p*NPα-l-Fuc, quantified by its absorbance at 405 nm. Enzyme reactions were performed by diluting the enzyme in 50 mM phosphate buffer, pH 7.0 at 37 °C and the reaction was started by the addition of *p*NPα-l-Fuc (10 mmol/L in reaction mixture). The reaction was stopped in 10 minutes by adding an equal volume of 10 % Na_2_CO_3_. One U is defined as the amount of the enzyme that releases 1 μmol of *p*-nitrophenol per minute.

### α-l-Fucosidase characterisation

Purified recombinant α-l-fucosidase was used for enzyme characterization. To measure Michaelis–Menten constant and maximal velocity, the chromogenic substrate *p*NPα-l-Fuc in concentration range 0.01-35 mmol/L in reaction mixture was used. The values of K_m_, K_s_ a V_lim_ were calculated using iterative method for statistical evaluation of deviations with the use of tools solver (Microsoft Excel). Following equation was used to calculate the constants, where v_0_ represents initial velocity, [S] substrate concentration, K_m_ is used for Michaelis constant, V_lim_ for maximum velocity and K_S_ is used for inhibition constant (Copeland 2000) [23]: ${v}_0=\frac{V_{lim}.\left[S\right]}{K_m+\left(1+\frac{\left[S\right]}{K_s}\right).\left[S\right]}$, taking into account the substrate inhibition.

Temperature optimum was determined by measuring α-l-fucosidase activity in different temperatures ranging from 20 to 70 °C. Temperature stability of the enzyme was determined by incubation of the enzyme at different temperatures (-20, 4, 30, 40, 50 and 60 °C) for specific time intervals and for subsequent standard activity assay. pH optimum of α-l-fucosidase was found by measuring the enzyme activity in a set of Britton–Robinson buffers (pH range from 2–11).

### Transglycosylation reactions

The ability of recombinant α-l-fucosidase iso2 to catalyse transfer of α-l-fucosyl moiety to different types of acceptor molecules was studied. All transglycosylation reactions were carried out in 50 mM phosphate buffer (pH 6.5) at 40 °C (25 mM EPPS buffer, pH 8 at 50 °C for the experiments with *p*-nitrophenyl glycopyranosides as acceptors). *p*NPα-l-Fuc served as a donor of l-fucose. It was solubilized in concentrated DMF, due to its low solubility in buffer. The final concentration of *p*NPα-l-Fuc in transfucosylation mixtures was 50 mmol/L (with the exception of the reaction mixture where *p*NPα-l-Fuc served as donor as well as acceptor and its final concentration was 83 mmol/L) and final concentration of DMF was 10 % (except of the mixture with *p*-nitrophenyl glycopyranosides as acceptors, where DMF was not used at all). The final concentrations of acceptors in a reaction mixture were: 33 mmol/L for *p*-nitrophenyl glycopyranosides (*p*-nitrophenyl α-d-galactopyranoside (*p*NPα-d-Gal), *p*-nitrophenyl α-d-glucopyranoside (*p*NPα-d-Glc) and *p*-nitrophenyl α-d-mannopyranoside (*p*NPα-d-Man), purchased from Sigma-Aldrich), 50 mmol/L for saccharides (d-glucose, d-galactose, d-fructose, d-mannose, d-maltose, d-glucosamine and N-acetyl-d-glucosamine, purchased from Sigma-Aldrich), 50 mmol/L for amino acid derivatives (*N*-(*tert*-butoxycarbonyl)-l-serine methyl ester (Boc-l-Ser-OMe) and *N*-(*tert*-butoxycarbonyl)-l-threonine methyl ester (Boc-l-Thr-OMe), purchased from Sigma-Aldrich), and 10 % for alcohols (methanol, ethanol, 1-propanol, 2-propanol, butanol, pentanol, octanol, purchased from Fluka). The amount of the enzyme in the reaction mixtures corresponded to 0.032 U/mL for reactions with *p*-nitrophenyl glycopyranosides, alcohols and amino acid derivatives and to 3.2 U/mL for reactions with saccharidic acceptors. Reaction time was 24 hours in all cases. After transglycosylation reactions the reaction mixtures were analyzed by TLC. Transglycosylation products of *N*-acetyl-d-glucosamine, methanol and ethanol were confirmed by mass spectrometry analysis in mode ESI+ (LTQ Orbitrap Velos, Thermo Scientific) with direct inlet in Central Laboratories of UCT, Prague. After removing of the enzyme by filtration (VectaSpin Centrifuge Tube Filter (12 kDa)), whole reaction mixtures were used for the analysis.

### Thin-layer chromatography

The transglycosylation reactions were analyzed on Silica gel TLC plates (Fluka) developed by a solvent system ButOH:EtOH:water (4:3:2, v/v/v) for p-nitrophenylglycopyranosidic acceptors, ethyl acetate: acetic acid: water (6:6:1, v/v/v) for saccharidic acceptors and ethyl acetate: acetic acid: water (7:2:2, v/v/v) for alcohols and amino acids used as acceptors. Saccharides were detected by 0.1 M 2-methylresorcinol (Alfa Aesar GmbH & Co KG, Germany) dissolved in 5 % (v/v) solution of sulphuric acid in ethanol (after heating of TLC plate).

## Abbreviations

Boc-l-Ser-OMe: *N*-(*tert*-butoxycarbonyl)-l-serine methyl ester
Boc-l-Thr-OMe: *N*-(*tert*-butoxycarbonyl)-l-threonine methyl ester
CAZy database: Carbohydrate-Active Enzymes database
DMF: *N*,*N*-dimethylformamide
IPTG: Isopropyl β-d-thiogalactopyranoside
LB: Luria-Bertani
*P*: *thiaminolyticus: Paenibacillus thiaminolyticus*
PCR: Polymerase chain reaction
*p*NPα-l-Fuc: *p*-nitrophenyl α-l-fucopyranoside
*p*NPα-d-Gal: *p*-nitrophenyl α-d-galactopyranoside
*p*NPα-d-Glc: *p*-nitrophenyl α-d-glucopyranoside
*p*NPα-d-Man: *p*-nitrophenyl α-d-mannopyranoside
SDS-PAGE: Sodium dodecyl sulfate polyacrylamide gel electrophoresis
TLC: Thin layer chromatography
X-Fuc: 5-bromo-4-chloro-3-indolyl α-l-fucopyranoside
